# Unrevealing sequence and structural features of novel coronavirus using *in silico* approaches: The main protease as molecular target

**DOI:** 10.17179/excli2020-1189

**Published:** 2020-03-17

**Authors:** Joseph Thomas Ortega, Maria Luisa Serrano, Flor Helene Pujol, Hector Rafael Rangel

**Affiliations:** 1 Department of Pharmacology and Cleveland Center for Membrane and Structural Biology, School of Medicine, Case Western Reserve University, Cleveland, OH 44106, USA; 2 Unidad de Química Medicinal, Facultad de Farmacia, Universidad Central de Venezuela, Caracas, Venezuela; 3 Laboratorio de Virología Molecular, Centro de Microbiología y Biología Celular, Instituto Venezolano de Investigaciones Científicas, Caracas, Venezuela

**Keywords:** Coronavirus, SARS-CoV-2, protease, treatment, HIV

## Abstract

Direct-acting antivirals are effective tools to control viral infections. SARS-CoV-2 is a coronavirus associated with the epidemiological outbreak in late 2019. Previous reports showed that HIV-1 protease inhibitors could block SARS-CoV main protease. Based on that and using an *in silico* approach, we evaluated SARS-CoV-2 main protease as a target for HIV-1 protease inhibitors to reveal the structural features related to their antiviral effect. Our results showed that several HIV inhibitors such as lopinavir, ritonavir, and saquinavir produce strong interaction with the active site of SARS-CoV-2 main protease. Furthermore, broad library protease inhibitors obtained from PubChem and ZINC (www.zinc.docking.org) were evaluated. Our analysis revealed 20 compounds that could be clustered into three groups based on their chemical features. Then, these structures could serve as leading compounds to develop a series of derivatives optimizing their activity against SARS-CoV-2 and other coronaviruses. Altogether, the results presented in this work contribute to gain a deep understanding of the molecular pharmacology of SARS-CoV-2 treatment and validate the use of protease inhibitors against SARS-CoV-2.

## Introduction

At present there is not specific treatment for coronavirus infection. However, prior outbreaks have helped to decipher the main pharmacological targets in these viruses. The therapies have been focused on targeting protease, helicase, polymerase, and using immunomodulators such as interferons and corticosteroids (Zumla et al., 2016[21]). Ribavirin alone or combined with IFN has been the most common therapeutic intervention in patients with SARS and MERS (Khalid et al., 2015[4]). Also, protease inhibitors have been used *in vitro* and *in vivo* to block coronavirus replication. Lopinavir (LPV) and ritonavir (RTV) are protease inhibitors currently used in HIV therapy that could block SARS-CoV and MERS-CoV main proteases (Savarino 2005[13]). The combination of lopinavir or ritonavir with ribavirin was associated with improvement in clinical outcome, compared with ribavirin alone, in SARS-CoV-infected patients (Lai, 2005[5]). During the MERS-CoV outbreak, the Food and Drugs Administration approved the use of ritonavir/lopinavir, based mainly on data obtained from *in vitro* studies (Sheahan et al 2020[14]). Altogether, these data support the assumption that some protease inhibitors may have an antiviral effect by blocking coronavirus main protease. However, like other RNA viruses, the main challenge associated with antiviral therapy is the selection of resistant variants. Mechanisms of generation of diversity in coronavirus are related to a moderate error rate of the polymerase (with proof-reading capacity) and homologous or heterologous recombination, factors that lead to antigenic drift and shift, similar to those described for Influenza viruses (Menachery et al., 2017[7]). Thus, viral replication will produce a diverse population of genome variants having different fitness profiles. These variants could be associated with the development of drug resistance (Yin and Wunderink, 2018[20]; Pruijssers and Denison, 2019[11]). The ongoing efforts toward discovering efficient drugs to prevent and treat SARS-CoV-2 infection should include the prior structural and pharmacological knowledge gained with the other coronavirus outbreaks. Based on that, in this work was established a comparative theoretical study to rationalize the potential use of protease inhibitors as a treatment against SARS-CoV-2 infections.

## Materials and Methods

### Sequence analysis

**Figure 1:**
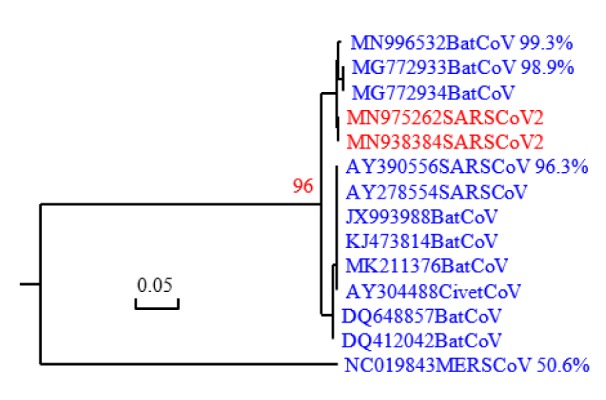
Phylogenetic analysis of SARS-CoV-2 and other coronaviruses main protease protein. Phylogenetic tree constructed with Poisson correction and 100 bootstrap replicas. The sequences are named with their accession number. The percent homology with SARS-CoV-2 protease protein is shown for some proteins.

Protein sequences of the main protease were individually retrieved from GenBank (accession numbers are shown in the phylogenetic tree, Figure 1(Fig. 1)) for SARS-CoV, SARS-CoV-2 and several Bat-CoV from the genus Betacoronavirus.

### Molecular docking

The coordinates for SARS-CoV and SARS-CoV-2 main proteases were obtained from the protein data bank, PDB code 1UJ1/2GX4 and 6LU7 respectively. Also, HIV-1 protease bounded to lopinavir under PDB code 1MIU and Bat HKU4 coronavirus PDB code 2YNB were evaluated. The PDB files to be used under further computational analysis were optimized by removing co-crystallized molecules and all crystallographic water molecules. Hydrogens were added and partial charges were assigned to all atoms. The obtained PDB files for each protein were further submitted to restrained molecular mechanics refinement. All molecular dynamics simulations described in this study were performed with NAMD 2.12 (Phillips et al., 2005[10]), Vega ZZ 3.1.0.21 (Pedretti et al., 2004[9]; Vanommeslaeghe et al., 2010[17]) as described in Ortega et al. (2019[8]). Following, structural analysis of the binding pocket was developed by using CASTp 3.0 software using the http://sts.bioe.uic.edu/castp/ server. The ligand-binding pocket located in the catalytic site was obtained manually and then verified by a priori docking approach with lopinavir by using the Achilles Blind Docking server (Sánchez-Linares et al., 2012[12]). The 3D structure of each inhibitor was obtained from PubChem. Also, public libraries for protease inhibitors were obtained from PubChem and ZINC databases. Molecular docking was performed with VINA/VegaZZ 3.1.0.21 and 30 runs conducted for each compound. The results were prioritized according to the predicted binding energy in kcal/mol. The results obtained from the docking simulation were visualized with the Biovia Discovery Studio Visualizer 17.2.0 software.

### ADME compound characterization

A comprehensive analysis of physicochemical descriptors, as well as ADME parameters, pharmacokinetic properties, drug-like nature, and medicinal chemistry for the top 5 compounds obtained from the library virtually screened, was carried out by using SWISSADME tools. These tools were asset through the website at http://www.swissadme.ch. 

## Results

### Homology sequence analysis of the main protease of SARS-CoVs and related Bat-CoVs

Phylogenetic analysis of the main protease protein sequences of SARS-CoVs and Bat-CoVs is shown in Figure 1(Fig. 1). The results are in agreement with recent reports of an independent introduction of SARS-CoV-2 from a Bat-CoV, different from the spillover which led to the introduction of SARS-CoV, being the Bat-CoV of *Rhinolophus affinis* the probable ancestor of this new virus (Wong et al., 2020[19]). The sequences of the whole main protease of this Bat-CoV and of SARS-CoV-2 share 99.3 % identity, while the MERS-CoV protease was only 50.6% identical (Figure 1(Fig. 1)). The main protease sequence is highly conserved among all the SARS-like coronaviruses from human and related CoVs from other animals (Figure 1(Fig. 1)).

### Structural analysis of the main protease

**Figure 2:**
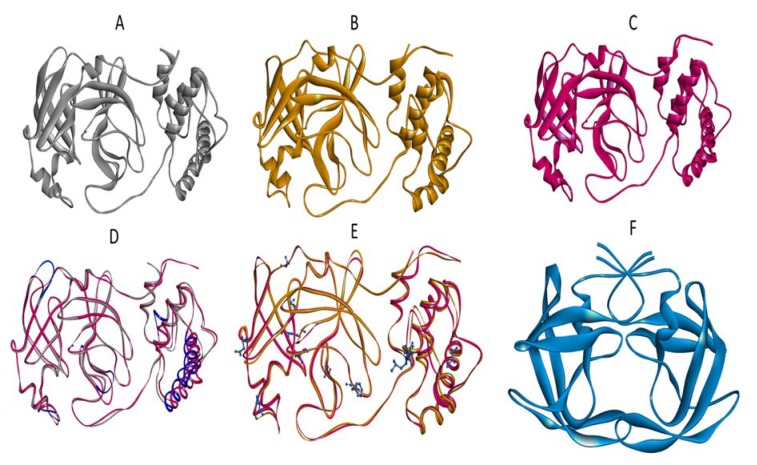
Coronavirus main proteases. Graphical representation for each protease was achieved in Biovia Discovery Studio Visualizer by using the coordinates obtained from the Protein Data Bank. Structure for A) Bat-CoV (2YNB), B) SARS-CoV (2GX4), C) SARS-CoV-2 (6LU7) are shown. Also, a comparison by molecular overlapping between these structures is shown in D. The main residues and changes in SARS-CoV-2 compared to SARS-CoV are shown in E. The HIV-1 protease (1MIU) is shown in F.

**Figure 3:**
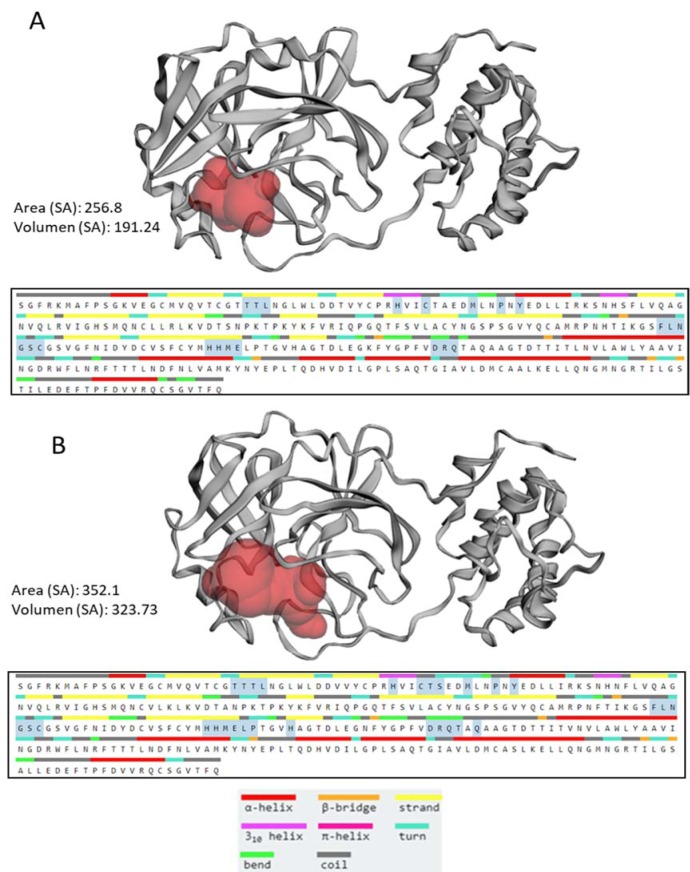
Coronavirus main proteases. Topological analysis of the catalytic site for SARS-CoV (A) and SARS-CoV-2 (B) main proteases. Conformational residues are highlighted in blue.

The SARS-CoV and SARS-CoV-2 proteins exhibit 96 % sequence identity to each other (Figure 1(Fig. 1)). Figure 2(Fig. 2) shows the 3D representation for Bat-CoV, SARS-CoV, and SARS-CoV-2 main proteases and indeed, very similar structures were observed, particularly when comparing SARS-CoV proteases, although very different to the one of HIV-1. Interestingly the SARS-CoV-2 protease shares 21/29 amino acids involved in the drug interaction with SARS-CoV protease, while this later exhibits only 21 amino acids involved in this interaction (Figure 3(Fig. 3)). SARS-CoV-2 protease has an active site with a surface accessible to solvent of 356Å and SARS-CoV only 256 Å (Figure 3(Fig. 3)).

**Figure 4:**
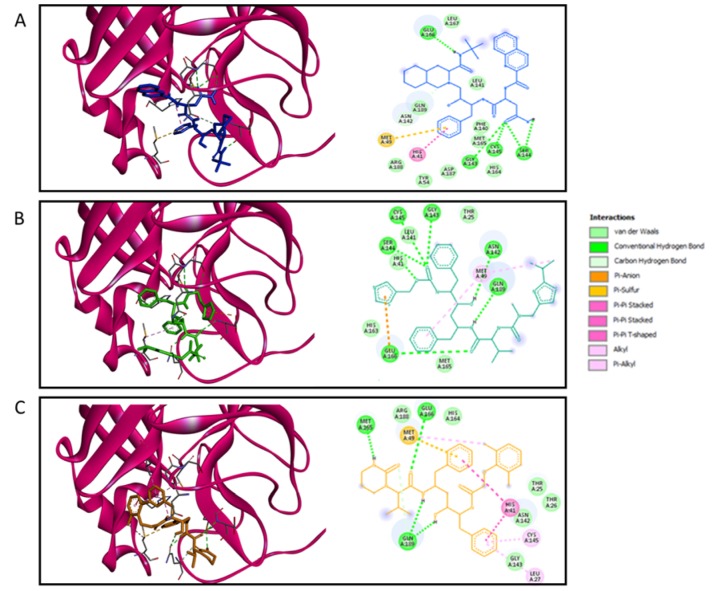
Molecular docking of HIV protease inhibitors on SARS-CoV-2 main protease. The molecular docking output represented as lower binding energy frame is shown for each inhibitor. 3D (left) and 2D (right) representations showing the main interaction between the inhibitor and the receptor are displayed. A) Saquinavir, B) Ritonavir, and C) Lopinavir

I*n silico *data demonstrated that the different protease inhibitors, used against HIV-1, could interact with the active site of SARS-CoV-2 protease producing an interaction with a binding energy lower than -6.9 Kcal/mol. However, the compound that produced the strongest interaction with the active site was saquinavir, with a binding energy of -9.6 Kcal/mol (Table 1(Tab. 1)). Binding energy of SARS-CoV-2 main protease to Saquinavir (SQV) and LPV were slightly higher, but similar, to the one of HIV-1 protease (Table 1(Tab. 1)). The interaction of LPV, SQV and RTV with the SARS-CoV-2 main protease is shown in Figure 4(Fig. 4), the different amino-acids involved in the interaction with all these drugs are also shown.

**Figure 5:**
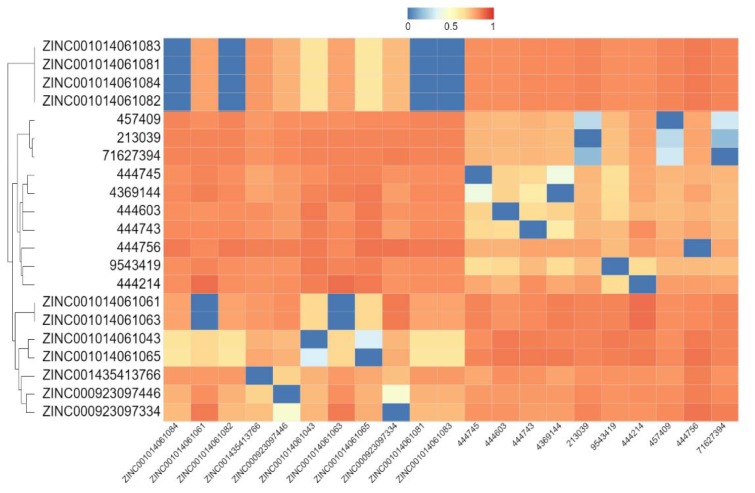
Hierarchical clustering of the top 20 best protease inhibitors obtained from public libraries (ZINC and PubChem) that could block SARS-CoV-2 protease. The compounds were clustered by chemical structure based in the Tanimoto coefficient where 0: the same structure and 1: no chemical relationship.

**Figure 6:**
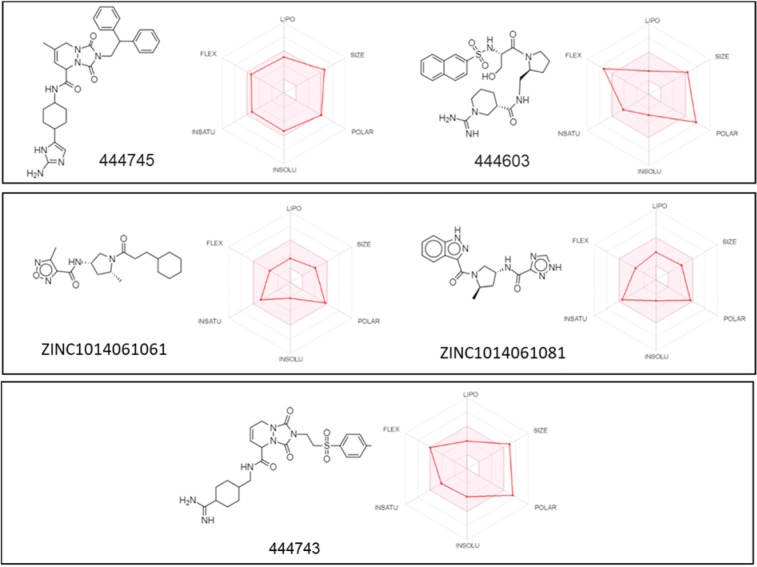
Chemical structure and ADME parameters of top five protease inhibitors obtained from public libraries (ZINC and PubChem) that could block SARS-CoV-2 protease. The colored zone is the suitable physicochemical space for oral bioavailability. LIPO (Lipophility): -0.7 <XLOGP3< +5.0. SIZE: 150 g/mol <MW <500 g/mol. POLAR (Polarity): 20Å2 <TPSA< 130Å2. INSOLU (Insolubility): 0< LogS (ESOL) <6. INSATU (Insaturation) 0.25< fraction Csp3 <1. FLEX (Flexibility): 0< Number of rotatable bonds <9

In order to contribute with further studies related to developing more effective drugs, in this work was evaluated a broad library of protease inhibitors available in the ZINC database (over 100 compounds) and PubChem (over 200 compounds). After molecular docking, compounds were prioritized based on binding energies. The top 20 compounds were clustered using a hierarchical matrix and represented into a heat map (Figure 5(Fig. 5)). These compounds could be clustered into three big groups based on their chemical structures. Also, the binding energies and chemical motives for each compound are shown in Table 2(Tab. 2). Interestingly, in the output of this virtual screening at least 5 compounds, representatives of these chemical clusters with binding energy around -8.2 Kcal/mol, were obtained. These scaffolds could be used as leading compounds to further chemical optimization to develop more potent inhibitors. The structure and main ADME parameters for each inhibitor are shown in Figure 6(Fig. 6), in general the 5 compounds shown, have physicochemical parameters under the expected values for a good drug.

## Discussion

Due to the rapid spread of SARS-CoV-2, affecting more than 70 countries, therapeutic alternatives are urgently required. Viral main protease plays a pivotal role in coronavirus replication. This enzyme is responsible for the cleavage of the polyprotein, producing functional proteins that will be packed into the virion. The molecular study of SARS-CoV-2 protease showed that this virus has high homology with SARS-CoV protease. Previous data suggest the use of protease inhibitors as potential inhibitors of SARS-CoV protease, in special some of those used for HIV-1 therapy. HIV-1 protease is an aspartyl protease, while in coronavirus it is a cysteine protease. However, the inhibition by protease inhibitors could be driven by a similar mechanism. HIV-1 is one of the best-studied models in antiviral research, their targets are well characterized and its inhibition by drugs has been broadly studied. Protease inhibitors play a main role in HIV-1 therapy. Furthermore, these compounds have shown inhibitory effects over proteases from pathogens such as Leishmania and malaria (Valdivieso et al., 2010[16]; Sonoiki et al., 2016[15]; de Wilde et al., 2014[1]). Thus, the use of protease inhibitors in other viral species could be supported by these non-specific protease inhibitions. Previous reports showed the inhibition of SARS-CoV protease by protease inhibitors used in HIV-1 therapy, being an interesting pharmacological target to treat SARS-CoV-2. Previously, scientific reports showed that some HIV-1 protease inhibitors could decrease SARS-CoV replication (Savarino, 2005[13]). Nevertheless, not all of the protease inhibitors showed the same efficacy against this virus (Jenwitheesuk and Samudrala, 2003[3]). Based on that, in this work, we established an *in silico* approach to validate the inhibition of SARS-CoV-2 protease by HIV-1 protease inhibitor. The results showed that the main protease inhibitors used in HIV-1 therapy could produce a decrease in the protease activity in coronavirus enzyme. The compound with the highest binding affinity was saquinavir. Preliminary reports are showing that patients infected with SARS-CoV-2 and treated with lopinavir could achieve viral clearance (Lim et al., 2020[6]). Our study suggests that drugs such as SQV and LPV have shown slightly higher, but comparative binding energies against the SARS-CoV-2, than HIV-1 protease. Thus, based on their safety profiles, SQV and LPV could be used as therapy and/or pre-exposure prophylaxis to reduce new infections by SARS-CoV-2. Additionally, the data obtained from prior outbreaks, the clinical results obtained by using protease inhibitors in patients infected with SARS-CoV-2, and the results shown here, support the use of protease inhibitors to treat SARS-CoV-2. 

In addition, to contribute to further development of antiviral drugs against SARS-CoV-2, in this work experimental compounds against the SARS-CoV-2 protease were evaluated by virtual screening. Some compounds showed comparable binding energy values to the SARS-CoV-2 protease than to those observed for SQV, LPV and RTV. ADME (for Absorption, Distribution, Metabolism, and Excretion) parameters estimation for new drugs, significantly helps to reduce the pharmacokinetics-related failure in clinical phases. These results evidence that these compounds could be suitable for further medicinal chemistry optimization to produce the second generation of inhibitors more specific and potent against SARS-CoV-2.

Other antivirals have been evaluated in SARS-CoV-2 as the polymerase inhibitor remdesivir, a nucleotide analog (currently in clinical trials against Ebola virus and SARS-CoV-2), alone or in combination with chloroquine, an inhibitor of lysosome acidification, with interesting results (Wang et al., 2020[18]; de Wit et al., 2020[2]). Therefore, proposing a combinatory therapy against SARS-CoV-2 could be a feasible approach. The starting therapeutic scheme could include remdesivir, with the disadvantage of its intravenous administration, and HIV-1 protease inhibitors such as Lopinavir or Saquinavir. Nevertheless, more pre-clinical and clinical data are required to support the development of new therapies against SARS-CoV-2.

## Figures and Tables

**Table 1:**
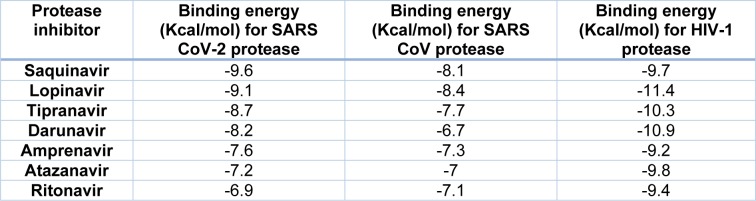
Molecular docking of Protease inhibitors used against HIV-1 over SARS-CoVs main proteases

**Table 2:**
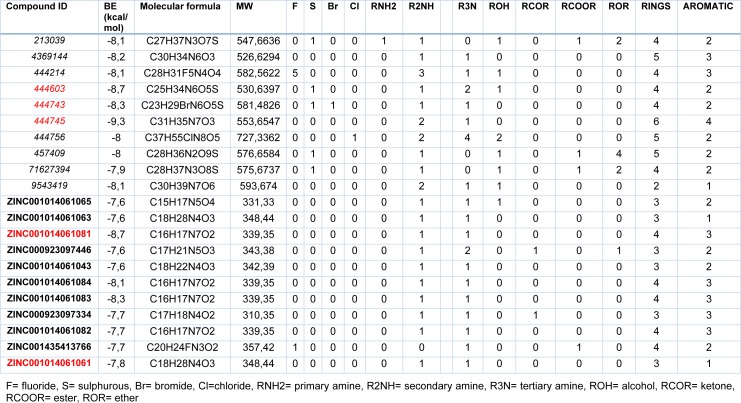
Binding energy (BE) values of the best 20 compounds selected as potential inhibitors of SARS-CoV-2 protease. Compound structures were obtained from ZINC database (Bold) and PubChem (italics), lowest BE compound of each group are shown in red.
